# Hospitals and the Poets.—II. The Medical Career of Oliver Goldsmith

**Published:** 1916-01-22

**Authors:** 


### HOSPITALS AND THE POETS.*

### II. The Medical Career of Oliver Goldsmith.

With the single exception of the present Poet-Laureate, to whom allusion was made in the previous article of this series, every poet who has engaged in medicine has abandoned it. Mr. Robert Bridges, having pursued his studies to the point of becoming a Fellow of the Royal College of Physicians, may be said more truly to have retired from practice with the laurels of science than to have exchanged them for the poet's bays. But the rule holds good with Goldsmith and Crabbe, two poets who engaged in medical studies with varying degrees of success before making their mark in their true pursuit—namely, literature. However, poetry and medicine were married in Goldsmith's poem "The Traveller," published in 1764, which bears the inscription "by Oliver Goldsmith, M.B.," on its title-page.

One of the minor controversies of scholarship has centred round the question whether Goldsmith ever really took a medical degree. The story that he did so was long thought to be as apocryphal as many incidents in his famous walking tour; but at all events the fact that he engaged in medical studies, and even went abroad to pursue them to the best advantage, was always admitted to be certain. In 1744, when he was sixteen years old, Oliver Goldsmith entered Trinity College, Dublin, where he was a contemporary of Edmund Burke, and, after some not too happy experiences, proceeded to Edinburgh in order to study medicine.

### Goldsmith as a Medical Student.

He left Dublin, however, a Bachelor of Arts, and Sir Ernest Clarke has expressed the view that he even began to study anatomy there before the interlude which ended with his arrival at Edinburgh in 1752. In January 1753 we find Goldsmith a member of the Students' Medical Society, and he seems to have been one of the pupils of Alexander Monro, then professor of anatomy. He next entertained the idea of completing his medical studies abroad, and his adventures began with his starting for Leyden in 1753. It was only after having to turn back to English shores that, on a second attempt, he succeeded in completing his journey. Once abroad, not much is known of the sequence of his movements, except that 1755 is the year in which his journey on foot began. Louvain, Paris, Strasbourg, Germany, Switzerland, and Italy are said to have been traversed by him as a solitary pilgrim, sometimes reduced to supporting himself by playing the flute along the road. It was either at Louvain or Padua that he was supposed to have taken his medical degree, until Sir Ernest Clarke, with the assistance of Sir William Osler, not long ago unearthed the following entry in the *Oxford Journal* of February 18, 1769: "Yesterday Oliver Goldsmith, Esq., Bachelor of Physick in the University of Dublin, Author of 'The Traveller: a Poem,' of 'The Present State of Polite Learning in Europe,' and of several other learned and ingenious Performances, was admitted to the same Degree in this University." How he became an M.B. of Dublin is a matter of conjecture, but Sir Ernest Clarke suggests that, as he was entitled under the statutes, Goldsmith on his return from his tour applied to the Dublin authorities to grant him the M.B. degree *in absentia*. Such a degree on certain conditions was obtainable by Bachelors of Arts like Goldsmith three years after graduation. The newspaper entry must lead us to suppose that this application was granted, and there is, therefore, no reason now to refer his medical degree to any foreign university, since Dublin and Oxford can both claim him as a graduate in medicine.

### An Unsuccessful Practitioner.

That he was an academic doctor on his return from abroad in 1756 is, however, unlikely: the first date of which we have evidence of Goldsmith subscribing himself "M.B." is 1763. But he was certainly endeavouring to practise before that date, and on his arrival in England, practically destitute, he became an assistant to a chemist at Fish Street Hill, met his friend Dr. Sleigh, and practised physic at Bankside, Southwark. That he was not very successful may be judged from the fact of his also obtaining a post as a school usher in the same year. The next year did not much improve his prospects either as a physician or a poet, for we find him writing to his brother that he is living "by very little practice as a physician, and very little reputation as a poet." So unpropitious were his fortunes that he even applied for what we should now call a medical officership to a factory on the coast of Coromandel! The final blow to his professional aspirations was given in the next year, when on December 21, 1758, he was examined at Surgeons' Hall for a certificate as hospital mate, but failed to satisfy the examiners. Like many another derelict of the learned professions, Goldsmith fell back on journalism, and four years afterwards laid the basis of his reputation by his "Chinese Letters," better known as "The Citizen of the World," which made his name on their appearance in 1762. In yet another four years his "Vicar of Wakefield" had appeared, and Goldsmith's sense of character and comedy was providing for his needs and illustrating his skill more successfully than his essays in medical practice. As a poet he lives in every well-stocked nursery as the author of the "Elegy on the Death of a Mad Dog"; and on his prose in essay, novel, and the drama there is equally little need to enlarge. Crabbe and other would-be physicians who became poets will be the subject of another article.

* The first article in this series appeared on page 329 of The Hospital for January 8.

